# How is “solidarity” understood in discussions about contact tracing apps? An overview

**DOI:** 10.3389/fpubh.2022.859831

**Published:** 2022-07-22

**Authors:** Max Tretter

**Affiliations:** Department of Systematic Theology, Friedrich-Alexander-Universität Erlangen-Nürnberg, Erlangen, Germany

**Keywords:** solidarity, contact tracing, digital tracing, COVID-19, SARS-CoV-2, review

## Abstract

**Background:**

In the context of the COVID-19 pandemic, there is much discussion about contact tracing apps, their use to contain the spread of the virus as well as the ethical, legal, and social aspects of their development, implementation, acceptance, and use. In these discussions, authors frequently mention “solidarity” when making key points in arguments. At the same time, authors rarely specify how they understand “solidarity”. This lack of specification about how they understand “solidarity” can lead to misunderstandings in discussions.

**Objective:**

To prevent such misunderstandings, it is important to specify how one understands “solidarity” when mentioning it in the discussions on contact tracing apps. Therefore, the aim of this paper is to elaborate how “solidarity” is understood in the context of contact tracing apps, i.e., how different authors understand “solidarity” when using it in discussions about these apps.

**Methods:**

In order to find out how different authors understand “solidarity” when discussing contact tracing apps, I conduct a literature review. I collect papers from several databases, inductively work out central differences and similarities between the different uses of “solidarity”, and use them to code and analyze relevant passages.

**Results:**

In the final sample, five different understandings of “solidarity” in the context of contact tracing apps can be identified. These understandings differ in how different authors (1) imagine the basic concept of solidarity, i.e., what “solidarity” refers to, (2) how they temporally relate solidarity to contact tracing apps, and (3) how they perceive the causal interactions between solidarity and contact tracing apps, i.e., the different ways in which solidarity and contact tracing apps influence each other.

**Conclusions:**

The five understandings of “solidarity” in the context of contact tracing apps presented here can serve as guidance for how “solidarity” can be understood in discussions—thus contributing to a better mutual understanding and preventing communicative misunderstandings.

## Introduction

During the COVID-19 pandemic digital contact tracing was introduced as a vital part of public health surveillance strategies. Countries (1–3) as well as private sector companies (4) develop and deploy their own apps for contact tracing. Such contact tracing apps (CTA) are typically used to collect and combine two sets of data: first, the user's contacts; second, their COVID-19 infection status. Contact information is often captured by collecting user location data either automatically *via* WiFi, cellular or GPS location data or manually *via* QR codes and transmitting it to central servers. There, they are subsequently analyzed by health authorities who identify contacts (3, 5). Alternatively, contact data can be collected through Bluetooth data exchange between smart devices. Typically, contacts are identified when two smart devices with compatible CTA are within a certain distance to each other for a certain period of time (5). If users are tested positive for COVID-19 or manually report an infection, their detected contacts often receive an anonymized exposure message saying that they have recently had contact with a person infected with COVID-19. Based on this data, some CTA may also display their users their risk of infection—for example, the German *Corona-Warn-App* shows whether the user is at none, low, or high risk of infection (6)—as well as provide guidance for managing potential exposure and infection (e.g., get themselves tested for COVID-19 or enter voluntary self-quarantine). The goal is to use CTA to detect at-risk contacts more quickly than through manual contact tracing and to be able to take action sooner (7), thus breaking chains of infection more effectively and containing the COVID-19 pandemic in the long term.

The effectiveness of CTA in combating COVID-19 depends centrally on how many people use CTA and are informed about risk contacts (8). Particularly because initial data suggest that “contact-tracing apps help reduce COVID infections” (9, 10) and are effective complementary tools for containing the spread of the virus (11–14) it is important to further increase their number of users to make them even more effective. This requires knowing the factors that drive their public acceptance or rejection in order to be able to adapt them (15, 16). While concern for one's own health and the prospect of more activity opportunities increase their acceptance, it is mainly privacy concerns and fear if government control that prevent their acceptance (15, 17). The latter can be countered by building and maintaining strong public trust (18)—just as these concerns can be solidified or increased by a lack of trust (19, 20). To preserve the former, it is important not to create overly optimistic expectations, which in the long term could lead to disappointment with CTA and subsequent loss of acceptance (5).

Digital contact tracing and CTA have already been used in other epidemiological events prior to the COVID-19 pandemic, e.g., the outbreak of Ebola in Sierra Leone in 2014–2016 (21, 22). During the COVID-19 pandemic, CTA are used for the first time worldwide. This stimulates many discussions about CTA, with one central issue being privacy (23, 24). From a legal and moral perspective, there is discussion about how much the state, public health authorities, or private institutions may monitor the movements (in the case of a GPS-based CTA) and contacts of individuals (25); how much this surveillance and invasion of privacy limits the freedom and autonomy of individuals (26); and how digital contact tracing relates to existing moral principles (27, 28) and legal data protection laws (29), particularly the GDPR (30). From a technical perspective, there is discussion about how to protect privacy best by asking what form of anonymization and encryption of data would be most effective (31) and whether to store data centrally or decentrally (32). Other questions are how high how high the acceptance rate needs to be (7) to guarantee the effectiveness of CTA (33); how the uptake of CTA could be increased (34, 35); whether the use of CTA should be legally mandated; and whether there is a moral obligation to use them (36). In addition, there is moral discussion about the principles and guidelines that should be implemented in CTA (27, 28) as well as sociological discussion concerning the public acceptance of CTA (37), and the expected social consequences of introducing CTA (38). These discussions about CTA are persistently relevant, because COVID-19 continues to spread and needs to be contained, but also because there may be future pandemics. In fact, the risk of pandemic outbreaks is currently higher than ever before, making future pandemics “inevitable” (39). It is therefore all the more important to be prepared for them and to be able to counter them effectively as soon as they break out (40). This preparation includes developing the necessary (contact tracing) technologies now and without pandemic constraints and time pressure (41), discussing and finding solutions to the relevant social, moral and legal issues now as well as establishing public trust in governments, institutions, and technologies now (42, 43)—to have these resources present and not to have to build them in the last minute when they are urgently needed.

While scoping the literature on CTA, I made two observations. First, the discussions often refer to “solidarity” in central points of the discussion. Especially when some of the moral or legal issues mentioned above are addressed. Solidarity, thus, seems to play an important role in legal, moral, societal, and public health discussions about CTA and captures a “spotlight” (44) in discussions about the COVID-19 pandemic. Second, authors rarely define how they understand the notion in their discussions, instead assuming that everybody intuitively understands what they mean when using “solidarity”. However, this assumption is deceptive and problematic. On the one hand, it is deceptive because no notion has a permanently fixed meaning and is understood the same way every time it is used. Instead, a notion acquires its meaning only when it is used and can only be understood by considering the context (45). As Bayertz (46) points out in his studies on solidarity, the same notion can even have contradictory meanings: “The concept of solidarity thus shares the same fate as other central concepts within ethical and political terminology, namely that of not being defined in a binding manner, and consequently of being used in very different and sometimes very contradictory ways” (46). On the other hand, it is problematic to assume that solidarity has a fixed meaning and not to specify how one understands the term when using it as this can lead to serious misunderstandings in discussions. Where two (or more) people use the same notion but understand it differently, one polysemous notion (47) can be understood in *multiple* ways and can become lexically ambivalent (48). This lexical ambivalence might in turn result in these people talking past, misunderstanding, and disagreeing with each other (49)—while in the worst case not even realizing it.

Especially when discussing important concerns as the fight against the COVID-19 pandemic and when using the notion “solidarity” at crucial points in arguments, it is important to avoid these communicative pitfalls. This means specifying exactly how one understands “solidarity” whenever one uses it and making clear for what purpose one is using it in the particular context. To help specifying the notion “solidarity”, I ask the research question: *what does solidarity mean in the context of the CTA*? Or, to be more precise: *how do different authors understand “solidarity” when using it in the context of CTA?*

Methodologically, I approach this research question by means of a literature review. After compiling a comprehensive sample, I analyze the passages that mention solidarity. By doing so, I identify central differences and similarities in their uses of “solidarity” and use these to elaborate different understandings of solidarity in the context of CTA. I then assign the papers to these understandings and thus work out which author understands solidarity in which way. Afterwards I discuss the *Results*, ask how these understandings of solidarity relate to each other, what limitations the study has, and finally give some practical recommendations.

## Methods

In conducting my review, I am guided by methodological frameworks for conducting a systematic review (50–52). In large parts, I follow the methodological approach for conducting a systematic review by Tranfield et al. (51). I search databases using a search strategy, then select the literature according to both external and internal inclusion criteria. Next, I inductively extract criteria from the literature, which I then use to examine how solidarity is understood in the sample. Details on how I proceed in searching, selecting and collecting data will now be presented.

To identify literature for my review I formulate a search strategy that is based on the keyword of the review question: what does *solidarity* mean in the context of *CTA*? The final search strategy consists of combinations of the keywords “contact tracing” or “tracing apps” in combination with the keyword “solidarity” (see Table 1) that are used to search the databases: *GIFT, Scopus, PubMed, Web of Science*, and *Google Scholar*. I search the databases at regular intervals and add the papers that were new since the last search to the sample. The first search was on January 07, 2021, and the last search was on December 21, 2021. During 2021, the GIFT database was discontinued by WHO for public use, so this database could not be included in the most recent searches. The results of previous searches in this database are retained in the final sample and not subsequently removed. The final sample includes publications from January 1, 2020 [when the first academic papers on COVID-19 were published (53)] to December 21, 2021 (when the final search was completed).

**Table 1:** Overview of the search, listing the names of the databases, the search strategies used, and the number of results.

**Database**	**Search strategy**	**# of results**
GIFT (until discontinued)	“Tracing apps” solidarity	17
	“Contact tracing” apps solidarity	41
Scopus	“Tracing apps” AND solidarity—TITLE-ABS-KEY	3
	“Contact tracing” AND apps AND solidarity—TITLE-ABS-KEY	1
Pubmed	“Tracing apps” [All Fields] AND solidarity [All Fields]	2
	“Contact tracing” [All Fields] AND apps [All Fields] AND solidarity [All Fields]	1
Web of Science	TOPIC: (tracing apps) *AND* ALL FIELDS: (solidarity)	1
	TOPIC: (contact tracing) *AND* ALL FIELDS: (apps) *AND* ALL FIELDS: (solidarity)	0
Google Scholar	“Tracing apps” solidarity	467

**Figure 1:**
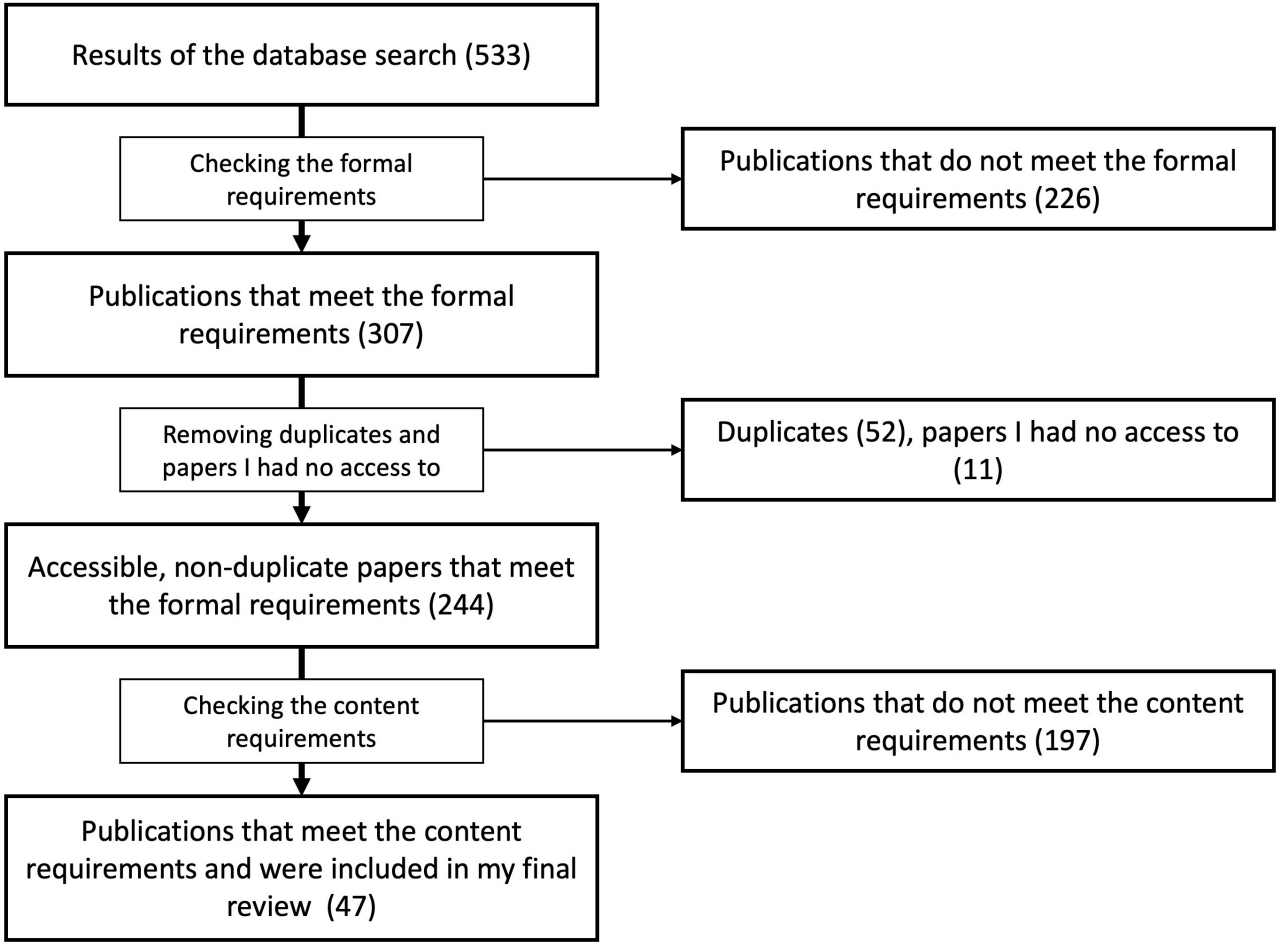
Procedure for selecting literature for my final review (created by the author).

The search strategy results in a corpus of 533 publications. Due to my language skills, I limit the review to articles written in English or German. Also, the review is limited to articles that are academic, already published, and reviewed to assure their quality (52). Based on these limitations pre-prints, gray literature, self-publications, student theses, and blog entries as well as publications in other languages are excluded (226)—leaving 307 journal articles, books, edited volumes, and articles in edited volumes that meet the formal requirements. After removing the duplicates (52) and the articles that I have no access to (11), I screen the remaining 244 full-text publications to examine whether they meet the content requirements to include them in my review or not (Appendix 1 in Supplementary Material 1). A paper is included in my final review if it mentions solidarity in connection with CTA. A paper is excluded if it does not make a connection between solidarity and CTA. The latter could be due to these papers using the notion “solidarity” as a proper name [e.g., *WHO's Solidarity Trial* in (54) or *France's Ministry of Solidarity and Health* in (55)] (22), mentioning tracing apps (56) or the notion “solidarity” (57) only in the references (58), or discussing both topics in different contexts without making a connection between them [e.g., when Brown et al. (59) discuss the challenges of immunity passports, they discuss the possibilities of a combining immunity certificates and CTA once and another time discuss the impact of immunity certificates on social solidarity—but both considerations are independent of each other] (60). After sorting out 197 papers, 47 papers are included in my final review (Appendix 1 in Supplementary Material 1). For a visual representation of procedure for selecting literature, see Figure 1.

The final 47 papers come from different disciplines (e.g., ethics, sociology, psychology, law, and tourism studies) and address different issues concerning CTA. I analyze them with a focus on the passages mentioning solidarity. The goal is to find out how different authors understand the “solidarity” when they use it in the context of CTA. Starting from a structuralist understanding of language (61), one must analyze the central differences (and similarities) between the various uses of the notion in the various papers and passages in order to work out how an author understands the notion “solidarity”. In short: to find out how an author understands “solidarity” in the context of CTA, one must show how her or his understanding differs from or relates to other authors' understanding of the notion.

In order to work out these differences and similarities between the various understandings of solidarity, comparative criteria are needed. As comparative criteria I use binary distinctions, which I call “key distinctions”. Each use of the term solidarity can be assigned to one side of the key distinctions. If two uses of the term solidarity are similar, they can be assigned to the same side of the key distinction; if they are different, they are assigned to different sides of the key distinction. Thus, differences and similarities between the different uses of the notion “solidarity” can be worked out. A binary distinction is a key distinction only if each use of the notion “solidarity” can be assigned to exactly one of its sides. There are several of these key distinctions. The more different key distinctions there are and the more often one use of the notion “solidarity” can be assigned to one of their sides, the more precisely one can work out its understanding of solidarity, i.e., the more precisely one can identify its differences and similarities to other uses of the notion “solidarity”.

The more key distinctions and the better they are, i.e., the more they help to make central differences visible, the more useful they are for elaborating the understanding of the uses of “solidarity”. Therefore, the central question is how to get specific key distinctions. There are two ways to get key distinctions: either one takes them from existing conceptual discussions of solidarity and uses them deductively to distinguish the understandings in the passages analyzed; or one or one extracts the key distinctions inductively from the passages that are being analyzed. In order not to pre-determine, limit, or bias the results by making prior assumptions about the notion's key distinctions (58) and to ensure that the key distinctions are tailored to the sample—i.e., they contribute as best as possible to making central differences visible—I choose the inductive approach. During several iterations I extract distinctions from the analyzed passages and test their validity on the other passages. I keep a distinction as key distinction if all analyzed passages could be assigned to one of its sides and discard or modify it if this does not work—thus arriving at the final key distinctions.

With these key distinctions, I code the passages mentioning solidarity and assign their use of “solidarity” to the corresponding sides of the different key distinctions. This assignment is based on criteria called “operators”. These operators act as rules for which side of the key difference a use of the notion “solidarity” should be assigned to. I extract these operators, like the key distinctions, inductively from the passages themselves in an iterative process. By coding the passages, I arrive at an understanding of how different authors understand solidarity in the context of CTA.

Based on the key distinctions, different understandings of the concept of solidarity in the context of CTA can be distinguished. The operators help to assign the different passages in which solidarity is mentioned to the different understandings and thus to answer the question: how do different authors understand “solidarity” when they use it in the context of CTA?

## Results

The sample first shows that different authors use “solidarity” with different degrees of precision: some authors mention the term only once as an “ethical buzzword” (62) without further specifying how they understand it, some authors define the term precisely and use it throughout. Second, it can be shown that different authors understand the notion “solidarity” differently in the context of CTA. In the sample, two key distinctions play a central role and help to distinguish the different understandings of “solidarity” in the context of CTA.

- The first key distinction I call “basic concepts of solidarity”: there are two different basic concepts of solidarity in the sample, which differ in how they understand solidarity and what they refer to.
- The second key distinction I call “temporal relations between solidarity and contact tracing apps”: there are two different ways in which solidarity temporally relates to CTA in the sample.
- In addition to the two key distinctions, two different “causal interactions between solidarity and CTA” can be distinguished, i.e., different ways in which solidarity and CTA interact with and influence each other in the sample.

In the results, I present these key distinctions—first, the different basic concepts; second, the different relations of solidarity and CTA—as well as the third distinction of different causal interactions. In doing so, I subordinate the distinctions to each other, i.e., the second key distinction is a sub-key distinction of the first key distinction and the different causal interactions are a sub distinction to the second key distinction.

### Basic concepts of solidarity

In the sample, one can first distinguish between two basic concepts of solidarity: Solidarity can either be understood as *a factual form of social cohesion*. As a factual form of social cohesion, solidarity describes the modes of how different individuals or groups of people live together (63) as well as the different bonds and organizational forms with which they structure their living together (46). Thus, as a form of social cohesion, solidarity always refers to existing collectives—and explores the interactions between CTA and the modes and organizational forms of their coexistence. Or solidarity can be understood as a *moral value*: something that people care about, that they consider good, and that grounds their judgment (64). As a moral value, solidarity comes into play in moral reasoning (65)—and can represent a principle used in considerations and discussions about CTA, a good used to guide them, or a criterion used to evaluate future or past considerations and discussions. Thus, as a moral value, solidarity always refers to considerations and discussions about CTA, and examines how this moral value affects them. Both basic concepts of solidarity are *not* mutually exclusive. Instead, the distinction helps to hermeneutically highlight different aspects of the notion “solidarity” that are in focus when it is used in a specific context—which I will elaborate further in Section Discussion.

If solidarity is understood as a factual form of social cohesion of a society, it can either refer to a specific, individual nation such as Singapore (66), Ireland (67), France (68), Germany (69, 70), South Korea (68), the UK (71, 72), and China (70) or even the European Union (73), or to an unspecified, individual nation (74–79). It can furthermore be an indeterminate collective of unknown size (36, 80–87), a particular marginalized group of persons within an indeterminate collective (38, 88–91), or an indeterminate human-technology-society called “post-digital hybrid assemblage” (92).

If solidarity is understood as a moral value, this moral value can be used in different discussions concerning CTA. It can be used when discussing or evaluating the development of CTA (28, 75, 80, 93–95), their implementation, i.e., their introduction into a society (95–100), or the organizational (101) or ethical (99, 102–106) framework of CTA. It can be used when individuals question themselves about whether to use CTA (107) or when individuals reflect on whether and how others use CTA (108). Solidarity can also be used when several of these points are discussed at once, e.g., the development and implementation (109) or the development, implementation, and use of CTA (110).

Looking at the absolute numbers, solidarity is more often understood as a factual form of social cohesion (29 papers) than as a moral value (22 papers). At the same time some papers understand solidarity as social cohesion *and* as moral value. For example, Lanzing understands solidarity both as the social cohesion that exists in an indeterminate country and as a moral value that guides decisions about the development of CTA (75). Leslie understands solidarity both as the social cohesion that exists in an indeterminate society and as a moral value that is used to guide the development of CTA (80).

### Temporal relations between solidarity and contact tracing apps

In the sample, one can then distinguish between two different ways in which solidarity temporally relates to CTA. It proves to be useful to maintain the above distinction of basic concepts when considering solidarity's relation to CTA. If solidarity is understood as a factual form of social cohesion, it can either precede or follow the acceptance and use of CTA in time. This distinction depends on whether the text examines the consequences of different factual forms of social cohesion for the development, introduction, acceptance, or use of CTA or, conversely, the consequences of the introduction, acceptance, or use of CTA for existing forms of social cohesion. As moral value, solidarity can be used to consider decisions about CTA from an ex-ante or ex-post perspective. This distinction depends on whether the decisions about CTA that the moral value refers to have already been made and are now being evaluated, or whether they are still pending and being discussed.

If solidarity is a form of social cohesion that precedes CTA in time, this form of social cohesion can be a condition for their public acceptance (67–71, 74, 76–78, 82, 85), a motivation for their individual use (36, 84, 85), or a necessary condition for their coordinated development (73).

If solidarity as a form of social cohesion follows CTA in time, this may have two different results. On the one hand, the public acceptance and individual use of CTA may strengthen the factual forms of social cohesion that exist in a collective (67) or strengthen factors that are elementary for them (72, 82), it may prevent negative consequences for forms of social cohesion that would have happened if CTA were not accepted and used (66), or open up possibilities for reimagining old and establishing new relationships (92). On the other hand, the acceptance and use of CTA may also have a negative impact on a society's forms of social cohesion: by threatening or undermining the forms of social cohesion that exist in a collective (83), by weakening factors that are elementary for them (75, 77, 80–82, 86), by reinforcing existing discriminations and worsening the situation for specific groups of people (38, 75, 90, 91), or by using resources for the development and implementation of CTA that would have had more positive effects on the community if used in an alternative way (88, 89).

As a moral value, solidarity can consider decisions about CTA—concerning their development, implementation into a society, or individual use—from an ex-ante perspective. Solidarity can guide or reflect the development of CTA morally (101, 106) and can prevent CTA from having negative impacts on their users (e.g., due to improper development) (75, 80, 93). Solidarity as a moral value can guide the introduction of CTA into a society morally (95, 98, 99, 104, 109), it can also motivate individuals to use CTA (72, 94, 107), and contribute to their public acceptance (110).

Similarly, solidarity as moral value can reflect on decisions about CTA that have already been made from an ex-post perspective, for example, by questioning past ethical discussions about CTA as to whether they have (sufficiently) taken into account the moral value of solidarity (28, 96, 102, 103, 105) or by evaluating how other people use CTA (108).

Looking at the absolute numbers, there are 16 papers in which solidarity as a factual form of social cohesion precedes CTA in time. There are 16 papers in which solidarity as a factual form of social cohesion follows CTA in time. There are 15 papers in which solidarity is used as a moral value to consider decisions about CTA from an ex-ante perspective. And there are eight papers in which solidarity is used as a moral value to reflect on decisions about CTA from an ex-post perspective. There are seven papers that relate solidarity and CTA in multiple ways at the same time. E.g., Gibney et al. (67) observe that the use of CTA at the same time “would benefit from and foster solidarity among the public in the national “figh” against the novel coronavirus” (9). Here, solidarity is understood as a form of social cohesion that precedes the use of CTA *and* results from their use.

### Causal interactions between solidarity and contact tracing apps

By means of these two key distinctions—the two basic concepts of solidarity and the temporal relations between solidarity and CTA—several understandings of solidarity in the context of CTA can be identified in the sample. Three understandings can be attributed one causal interaction between solidarity and CTA, i.e., one particular way in which solidarity influences the development, use, or acceptance of CTA or decisions regarding them. Two different ways of causal interaction between solidarity and CTA can be attributed to the understanding of solidarity as a form of social cohesion that follows CTA in time.

- If solidarity is understood as a form of social cohesion that precedes CTA in time, it can be a crucial condition for the implementation (67, 76) of CTA or encourage their public acceptance (67, 69–71, 76, 78, 79, 84, 85). See, for example, Milne and Costa (68) who describe solidarity as condition for the implementation and acceptance of CTA: “For example, CTA, such as those now implemented in France, Germany or South Korea, rely on adoption through a sense of solidarity”. In addition, solidarity itself or appeals to a “we are all in this together” sense of social cohesion can motivate single individuals to use CTA, argue Parker et al. (36).
- If solidarity is understood as a form of social cohesion that follows CTA in time, the implementation and use of the latter can reinforce the former. As Gibney et al. argue, the use of CTA could give individuals a common sense of working together to fight the battle against COVID-19 (67), or, as Samuel and Sims put forward, it could provide them with shared visions or impose equal social obligations (72). Both, according to the authors, could constitute, maintain, and strengthen forms of social cohesion itself or factors that are elementary for it. Conversely, the use of CTA can also prevent negative consequences for social cohesion that might have occurred if hey were not implemented and used (66). Furthermore, CTA can open up possibilities of reimagining old and establishing new relationships and create new forms of social cohesions, e.g., by creating new, digital bonds between actors who previously had no contact with each other (92).
- If solidarity is understood as a form of social cohesion that follows CTA in time, the implementation and use of the latter can weaken or undermine the former (86, 90). E.g., as Pila (83) suggest, by providing “too much information about individuals' health risks” CTA “can undermine solidarity by depriving people of the very uncertainty about their own and others' fates on which a commitment to sharing those fates depends.” This can result in existing vulnerabilities (75) or marginalizations being reinforced (38, 91). E.g., as Chang et al. point out, by warning CTA users not to have contact with homepless persons and drug users for health reasons—thereby adding another stigma to these groups of people (38). Alternatively, the resources used for the development and implementation of CTA could have had more positive effects on the community if used in an alternative way (88, 89).
- If solidarity is understood as a moral value that considers decisions on CTA from an ex-ante perspective, it can guide and orient decisions or actions concerning the development (75, 80, 102, 106, 109), implementation (75, 95, 98, 99, 104, 110), and use (94, 102) of CTA ethically. For example, Leslie advocates taking into account the UK Government's SUM values for safe and ethical AI, which include solidarity, when developing CTA (80), and Gasser et al. elaborate a diagram of six different values, one of which is solidarity, from which they then derive 17 moral challenges to be considered in the development of CTA (109). Also, educating individuals about the moral value of solidarity, as Roche notes, can help them become more aware of their role in fighting the virus (107).
- If solidarity is understood as a moral value that considers decisions on CTA from an ex-post perspective, it can serve as an ethical criterion for the evaluation of past discussions or decisions concerning the development (96, 102, 103) as well as the implementation (96, 97, 100, 102, 103, 105) of CTA. For example, Keating reflects on whether collaboration during the development and implementation of CTA would have worked better if solidarity had played a more central role (97), and Siffels wonders whether it would have been better to focus questions about CTA development and implementation not only on privacy and health, but also to consider other values, including solidarity (103). Furthermore, solidarity can be used as a criterion to evaluate other people's behavior toward CTA (108), when people consider other people's decision to use CTA as solidary or their decision not to use it as unsolidary (108).

The causal interactions assigned are not key distinctions, as they do not apply a binary distinction to all uses of the notion “solidarity” in the sample. Rather, these causal interactions specify the understandings of solidarity identified in the sample. This specification leads to adding a binary distinction to the second understanding, so that we can ultimately speak of five understandings of solidarity in the context of CTA.

### Illustration of the five understandings of solidarity as a tree diagram

**Figure 2:**
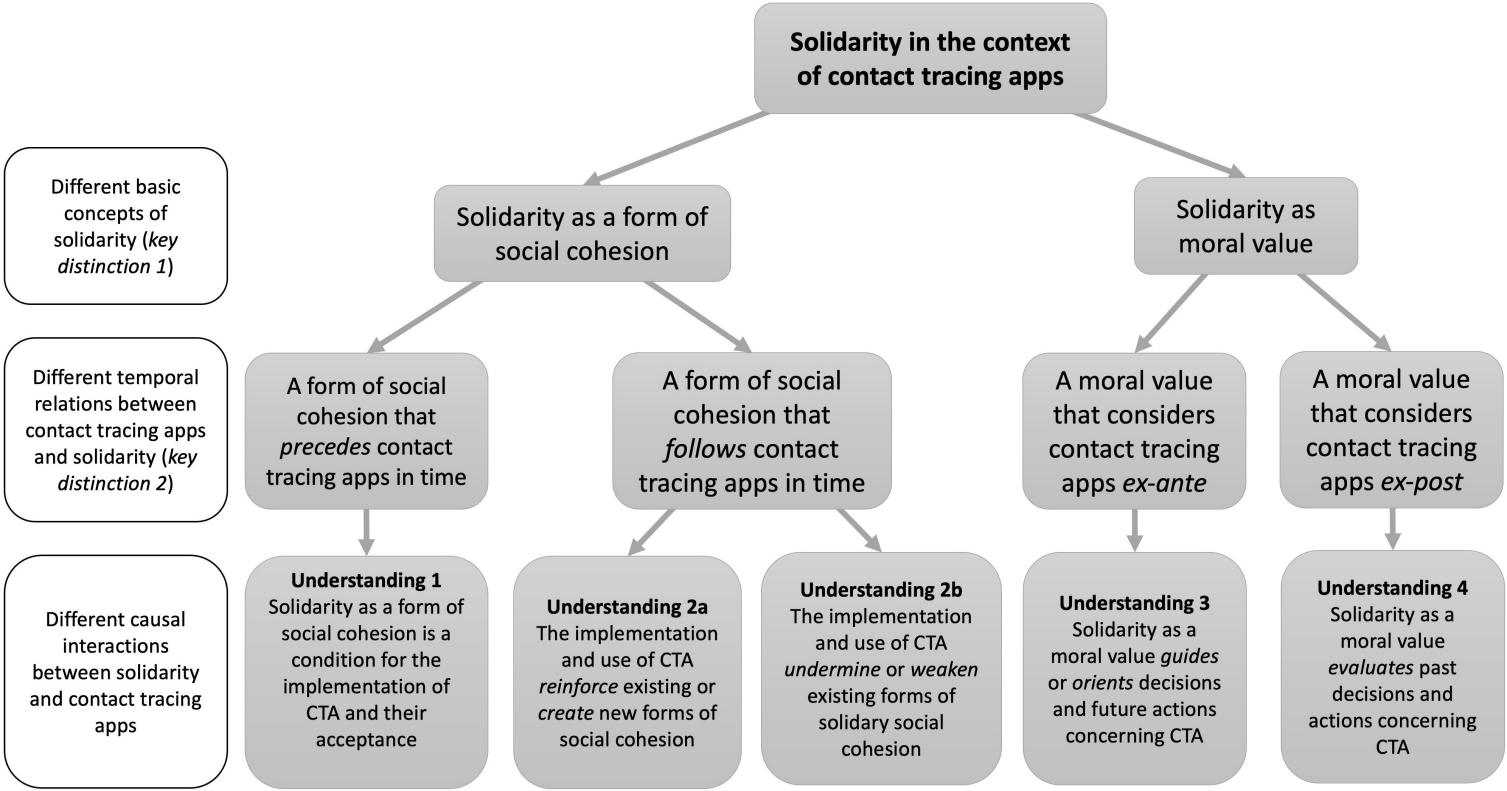
Illustration of the five understandings of solidarity in the context of CTA (created by the author).

Given these two key differences and the different causal interactions between solidarity and CTA, there are five different understandings of solidarity in the context of CTA. These five understandings, as well as the distinctions that lead to them, can be illustrated as a tree diagram (see Figure 2). Starting from the general notion of “solidarity in the context of CTA” one can apply the first key distinction and distinguish between the two basic concepts of solidarity, then one can apply the second key distinction and distinguish between the different temporal relations of solidarity and CTA, thirdly one can further specify by adding the different causal interactions between solidarity and CTA.

### Assignment of the papers to the five understandings

Along this tree diagram, all 47 papers can be assigned to (at least) one of these understandings. To do so, one must first decide whether a paper understands solidarity as a form of social cohesion or as a moral value. There is a list of operators that indicates whether solidarity is understood as the former of the latter. Subsequently, one can use the next list of operators to assign each paper to (at least) one understanding of solidarity.

Operators indicating that solidarity is understood as a form of social cohesion are: Solidarity explicitly refers to a social system (e.g., societies and communities); solidarity denotes an organizational form of social systems (e.g., nations, countries, the public sphere, and community initiatives); solidarity refers to groups or individuals of social systems (e.g., poor people, citizens of a state, the public, and population); solidarity describes the cohesion of a social system or an essential factor for it (e.g., shared feelings); solidarity describes a group of persons close to each other (e.g., families); solidarity describes the cooperation of different actors; or solidarity describes a relationship between different actors (e.g., human and technology). Operators indicating that solidarity is understood as a moral value are: Solidarity is explicitly described as a moral value or ethical principle; solidarity is mentioned in a series with other moral values or ethical principles; solidarity is described as belonging to the good; Solidarity is mentioned in reference to concerns, considerations, decision, evaluations or failures; or solidarity is taught through moral education.

Subsequently, the second key distinction and the causal interactions—which I have summarized in the following list of operators for the sake of clarity—can be used to classify how solidarity relates to CTA in terms of time and causality.

- If solidarity is understood as a form of social cohesion, operators indicating that solidarity precedes CTA in time are (*understanding 1*): Solidarity is described as condition for the implementation and acceptance of CTA; solidarity promotes the acceptance and use of CTA; or solidarity is necessary for the development of CTA.
- If solidarity is understood as a form of social cohesion, operators indicating that solidarity follows from CTA and that CTA constitute or strengthen solidarity are (*understanding 2a*): The text explicitly states that CTA constitute or strengthen social cohesion; CTA strengthen factors that are elementary for social cohesion; CTA prevent negative consequences for the social cohesion; or CTA open up possibilities of reimagining old and establishing new relationships.
- If solidarity is understood as a form of social cohesion, operators indicating that solidarity follows from CTA and that CTA weaken or undermine solidarity are (*understanding 2b*): The text explicitly states that CTA threaten, weaken or undermine social cohesion; CTA weaken factors that are elementary for social cohesion or reinforce factors that damage social cohesion; the introduction of CTA worsens the situation of a group of people or increases existing vulnerabilities or marginalizations; or the resources used for the development and implementation of CTA would have had more positive effects on the community if used in an alternative way.
- If solidarity is understood as a moral value, operators indicating that solidarity considers CTA from an ex-ante perspective are (*understanding 3*): Solidarity is referred to as a central criterion for ethical or legal concerns, considerations or decision; solidarity is introduced to orient or guide a decisions or actions; solidarity imposes obligations to act; or solidarity helps individuals to become aware of their role.
- If solidarity is understood as a moral value, operators indicating that solidarity considers CTA from an ex-post perspective are (*understanding 4*): Solidarity refers to past discussions or decisions; solidarity serves as criterion for the evaluation of past discussions or decisions; solidarity is presented as a moral value with which past failures could have been avoided; or solidarity is a criterion to evaluate other people's behavior.

Since the list of operators in the text can be somewhat confusing, there is a table of the various understandings of solidarity and their corresponding operators in Appendix 2 (Supplementary Material 2, starting from page 1).

Using the operators to identify how “solidarity” is understood in the passages mentioning the notion, one can assign the papers to the understandings. A paper is assigned to an understanding if the operators indicate that solidarity is understood in this way in at least one passage of the paper. A paper can also be assigned to several understandings if the operators identify different understandings in several passages of a paper. A detailed overview of the passages analyzed and their assignment to an understanding of solidarity in the context of CTA using the above operators can be found in Appendix 3 (Supplementary Material 2, starting from page 3).

As Table 2 with the overview of the different understandings and the authors shows, the five understandings of solidarity in the context of CTA occur with different frequency in the sample: most frequently, the notion “solidarity” is understood as a form of social cohesion that precedes CTA, is a condition for their implementation, and encourages their public acceptance and individual use (*understanding 1*, 16 times), closely followed by the understanding of solidarity as a moral value that considers CTA from an ex-ante perspective that orients and guides decisions about future actions concerning CTA (*understanding 3*, 15 times). Third most common is the understanding of solidarity as a form of social cohesion that is undermined or weakened by the implementation and use of CTA (*understanding 2b*, 12 times). Less frequently, the notion “solidarity” is understood as a moral value that considers CTA from an ex-post perspective, reflects and evaluates past decisions concerning their development, implementation, and use (*understanding 4*, 8 times), or as a form of social cohesion that is reinforced or created by the implementation and use of CTA (*understanding 2a*, 5 times).

**Table 2:** Overview of the different understandings and the authors.

**Understanding 1**	**Understanding 2a**	**Understanding 2b**	**Understanding 3**	**Understanding 4**
Dowthwaite et al. (69)	Gibney et al. (67)	Alemanno and Bialasiewicz (90)	Batifoulier and Diaz-Bone (95)	Braun and Hummel (28)
Georgieva et al. (74)	Lee and Lee (66)	Barocas et al. (91)	Blauth and Gstrein (104)	Christofidou et al. (105)
Gibney et al. (67)	Nijsingh et al. (82)	Chang et al. (38)	El-Haddadeh et al. (106)	Hendl et al. (96)
Lu et al. (70)	Price (92)	French et al. (88)	Findlay and Remolina (93)	Hoffman et al. (102)
Matt (84)	Samuel and Sims (72)	Lanzing (75)	Gasser et al. (109)	Kaspar (108)
Milne and Costa (68)		Leslie (80)	Hoffman et al. (102)	Keating (97)
Montanari Vergallo et al. (85)		Mangan et al. (81)	Hummel and Braun (101)	Siffels (103)
Nanni et al. (76)		Milan (89)	Kahn (110)	Watson et al. (100)
Nijsingh et al. (82)		Nijsingh et al. (82)	Lanzing (75)	
Parker et al. (36)		Pila (83)	Leslie (80)	
Sagan et al. (78)		Sekalala et al. (77)	Mbunge et al. (98)	
Samuel et al. (71)		van Hees et al. (86)	Mbunge et al. (99)	
Saunes et al. (79)			Mello and Wang (94)	
Sekalala et al. (77)			Roche (107)	
Stefan (73)			Samuel and Sims (72)	
Wnuk et al. (87)				

The authors that are assigned to multiple understandings are highlighted gray.

## Discussion

The results show that there are five different understandings of solidarity in the context of CTA in the sample. These different understandings differ in how authors determine the basic concept of solidarity (solidarity as a form of social cohesion or as a moral value), the temporal relation between solidarity and CTA (solidarity either precedes or follows CTA) and the causal interactions between solidarity and CTA. The five different understandings vary in frequency in the sample. These results raise further questions: First, how do the different understandings of solidarity in the context of CTA relate to each other—are they mutually exclusive or complementary? Second, are the key distinctions on which these different understandings are based valid—i.e., are they really five *different* understandings of solidarity in the context of CTA? Third, what is the scope of these findings and what are their limitations? And fourth, how can these results help address the communication problems mentioned in the *Introduction*?

### Relations between the five understandings of solidarity

These five different understandings of the notion “solidarity” in the context of CTA can relate to each other in different ways.

On the one hand, there are a total of seven papers out of 47 papers—and thus a significant portion—which can be assigned to more than one understanding of solidarity. Assuming that these papers are not incoherent in how they understand or use the notion “solidarity” and do not contain self-contradictions, these seven papers suggest that the five different understandings do not need to be contradictory. Instead, the different understandings can stand side by side, indicating that they are mutually complementary. For example, Nijsingh, van Bergen, and Wild describe the relationship between solidarity and CTA as feedback loops: “Attempts to responsibly introduce CT technology are thus confronted with *feedback loops*: *low effectiveness raises costs and decreases uptake*, attempts to counter this by raising effectiveness may decrease privacy, which then potentially decreases uptake, while raising uptake by implementing more or less mandatory approaches creates risks of backlash and crumbling public support, which then again lowers effectiveness. Of course, scenarios where positive reinforcing feedback loops take place are also possible. A fair and reliable system would lead to an *increase in trust and potentially a shared feeling of solidarity*, which will lead to a further increase in use and therefore effectiveness, etc.” (82) (italics added). When they describe solidarity as a shared feeling, they understand it, as the wider context of the passage makes clear, as an essential factor for the cohesion of a social system. The effective use of CTA can contribute to increasing this form of solidarity (*understanding 2a*), just as, conversely, the non-effective use of CTA can lead to a decrease in it (*understanding 2b*). At the same time, a circular relationship between CTA and solidarity as a feedback loop implies that solidarity contributes to the acceptance of CTA (*understanding 1*). A second example is Samuel and Sims and their accounts of solidarity and CTA: “Our findings showed how, through the mixture of both promissory discourses and altruistic discourses of *solidarity, an imaginary* was created that was imbued with implicit understandings of what is good or desirable in the social world. The *future-oriented visions and promises attached to the app*, along with calls of *social obligation*, constructed the trial of the app as a venture which was *morally good*, which was valued because of its ability to bring health benefits, and which was desirable in *the social world of the Isle of Wight*” (72) (italics added). They present solidarity as a currently imagined, future form of social cohesion in a particular social environment. The use of CTA, according to these visions, has a positive effect on social cohesion itself or central factors for it (*understanding 2a*). At the same time, solidarity is presented as a moral value that belongs to the morally good and from which a social obligation to use CTA arises (*understanding 3*). The understanding of solidarity as a moral value thereby emerges from the understanding of solidarity as an imagined form of social cohesion. In both Nijsingh, van Bergen, and Wild and Samuel and Sims, different understandings of solidarity in the context of CTA simultaneously coexist and complement each other.

On the other hand, the different understandings of solidarity can also contradict each other and lead to opposing recommendations. This becomes evident, when contrasting how, e.g., Kahn (110) understands “solidarity” with how Chang et al. (38), or how Pila (83) understand the notion. Kahn (110) assumes that the implementation and use of CTA will have a positive effect on society by counteracting the spread of COVID-19. Accordingly, he favors CTA and uses the notion “solidarity” to promote their implementation and use (*understanding 1*). In contrast, Chang et al. (38) and Pila (83) demonstrate how the introduction of CTA has negative effects on society or individual groups within it. They use “solidarity” to show how CTA can undermine it and to warn against the use of CTA in the name of solidarity (*understanding 2b*). These authors' examples show how different understandings of solidarity in the context of CTA can contradict each other and lead to different recommendations.

Contrasting both positions shows that the different understandings of solidarity in the context of CTA can both complement but also contradict each other—and even lead to opposing recommendations. This makes it even more important to define exactly what the notion means whenever it is used.

### The hermeneutic nature of the key distinctions

One result of the review is that in the context of CTA two basic concepts of solidarity can be distinguished. Solidarity is understood either as a factual form of social cohesion *or* as a moral value—this key distinction is the basis of the five understandings of solidarity.

At first glance, this distinction seems to be contradictory to the concept of solidarity, as it is outlined in the discussions about solidarity. As, e.g., Bayertz (46) shows in his study Four *uses of “solidarity”*, this distinction cannot be strictly maintained. He refers to the history of the concept solidarity and shows that only the earliest academic uses of solidarity understand it *solely* as a factual form of social cohesion. Yet, already Émile Durkheim at the end of the nineteenth century presents a concept of solidarity, that includes both, factual *and* moral aspects. For him, solidarity primarily refers to the various ties that bind members of a society together and thus ensure its cohesion (111). But he continues to show that this social cohesion has considerable influence on morality and moral obligations of the community (112)—thus indicating that factual forms of social cohesion and moral values can never be separated from each other (113). Durkheim's contemporaries, as Bayertz (46) and ter Meulen (114) show, share this understanding of solidarity. Bourgeois (115), for instance, uses the notion “solidarity” to refer to the factual forms of social cohesion in a society *as well as* to describe the moral obligations that arise from it. This understanding of solidarity as both factual form of social cohesion *and* moral value, prevails, so that: “‘Solidarity’ is now comprehended as a mutual attachment between individuals, encompassing two levels: a *factual* level of actual common ground between the individuals and a *normative* level of mutual obligations to aid each other, as and when should be necessary.” (46).

This understanding of solidarity as a moral value, which is based on factual forms of social cohesion and aims at maintaining the community through its members solidary supporting each other, is common sense today in almost all publications concerned with solidarity (60, 114, 116–120). While there are still different understandings of how a solidarity community is constituted, what forms of cohesion exist within it, and what moral values arise from it, the simultaneity and mutuality of moral values and factual forms of social cohesion is set.

When I speak of solidarity either as a factual form of social cohesion or of solidarity as a moral value, I do not intend to fall behind the state of research and do not wish to separate and isolate moral values and factual forms of social cohesion from each other. By distinguishing between these two basic concepts, I want to do justice to the observation that many authors in the sample, when they use “solidarity”, put an emphasis—and they understand solidarity either *rather* as a factual form of social cohesion or *rather* as a moral value. The distinction between both basic concepts should help to identify these emphases *in a hermeneutic way*—without separating factual forms of social cohesion and moral values or ignoring their reciprocity.

If the underlying key distinction is already hermeneutic in nature and indicates different emphases without introducing a strict separation between two basic concepts, the resulting five understandings of solidarity are also hermeneutic in nature—and serve to capture the various aspects, nuances and emphases of “solidarity” in the context of CTA.

### Limitations

The review shows how “solidarity” is understood by different authors in the context of CTA. It proceeds inductively and is oriented toward the passages that mention solidarity in the context of CTA. Still—or maybe due to this approach—the review has some limitations.

First, the results are based on a relatively small database of only 47 papers, which are included in the final review. The database could be expanded if non-reviewed, non-academic papers, and gray literature were included. But this would, in turn, reduce the average assessed quality of the papers (52). So, although the database is small, it is of high quality. Second, the question about the different understandings of solidarity in the context of CTA is very specific—it focuses on only one notion in one single context. However, the same applies here: precisely due to its narrow focus, the review is highly informative.

Both limitations raise the question of whether the results are valid beyond this one context. On the one hand, it must be noted that the five understandings of solidarity as elaborated here are only valid for the context of CTA. On the other hand—as shown not least by the above discussion on the distinction between the two basic concepts—some of the distinctions made here can be found elsewhere in the discussions on the concept of solidarity. Thus, while the five understandings of solidarity have no validity beyond the context of CTA, they offer representative distinctions within the concept of solidarity that also prove to be valid elsewhere.

### Practical recommendations

In conclusion, some practical recommendations can be given regarding the different understandings of the notion “solidarity” in the context of CTA. First, it is important to specify precisely how one understands “solidarity” when using the notion in academic (or political) discussions about CTA. This helps to avoid polysemy, lexical ambiguity, and talking past each other, and thus contributes to preventing the central communicative misunderstandings identified in Section Introduction—as well as the negative consequences that result from them. The five understandings of “solidarity” in the context of CTA, presented in Section Results, can serve as orientation and help specify the notion in discussions.

Second, communication problems and misunderstandings also limit the success of public health communication (121, 122). Therefore, when using the term “solidarity” in public health communication, it is important to be specific about how it is understood. Because failing to do so, and not being clear about how and why the notion “solidarity” is used to promote public health intervention, e.g., the use of CTA, may in the worst case lead, as Guttman and Lev show (123), to the public beginning to distrust the notion—as well as the goals or public health measures it promotes and the institutions that use it. To prevent this loss of public trust, it is important not to strain the notions in public health communication (124) and to use them as clearly as possible—which, in the case of solidarity and CTA, the five understandings in Section Results can help to do.

## Data availability statement

The original contributions presented in the study are included in the article/supplementary material, further inquiries can be directed to the corresponding author/s.

## Author contributions

The author confirms being the sole contributor of this work and has approved it for publication.

## Funding

This work has been funded by a Grant from the Federal Ministry of Research and Education (Grant Number: 01GP1905B). The funder played no role in conducting the research or writing the paper.

## Conflict of interest

The author declares that the research was conducted in the absence of any commercial or financial relationships that could be construed as a potential conflict of interest.

## Publisher's note

All claims expressed in this article are solely those of the authors and do not necessarily represent those of their affiliated organizations, or those of the publisher, the editors and the reviewers. Any product that may be evaluated in this article, or claim that may be made by its manufacturer, is not guaranteed or endorsed by the publisher.
