# Supplementary material for: How is “solidarity” understood in discussions about contact tracing apps? An overview
Source: Front Public Health. 2022 Jul 22;10:859831. doi: 10.3389/fpubh.2022.859831 (PMC9355132; doi:10.3389/fpubh.2022.859831)
Supplement: Supplementary file 2 [file Data_Sheet_1.docx]

Supplementary Material

# Appendix 2

The following table is an illustration of the of the explanations in chapter 3.5 *Assignment of the papers to the five understandings*. It illustrates the different operators that can be used to assign a mention of CTA in the text to an understanding. The key differences of whether solidarity is understood as a form of social cohesion and as a moral value as well as the different understandings of solidarity are highlighted in gray, below them and highlighted in white are the operators that can be used to assign a mention of solidarity to this key difference. To assign the mention of solidarity in a paper to (at least) one understanding, one must first decide, using the first list of operators, whether a paper, or a passage in a paper, understands solidarity as a form of social cohesion or as a moral value. Subsequently, one can use the next list of operators to assign each paper to (at least) one understanding of solidarity.

| **Solidarity** | | | | |
| --- | --- | --- | --- | --- |
| **Solidarity as a form of social cohesion** | | | **Solidarity as a moral value** | |
| Solidarity explicitly refers to a social system (e.g., societies, communities);  solidarity denotes an organizational form of social systems (e.g., nations, countries, the public sphere, community initiatives);  solidarity refers to groups or individuals of social systems (e.g., poor people, citizens of a state, the public, population);  solidarity describes the cohesion of a social system or an essential factor for it (e.g., shared feelings);  solidarity describes a group of persons close to each other (e.g., families); solidarity describes the cooperation of different actors;  or solidarity describes a relationship between different actors (e.g., human and technology). | | | Solidarity is explicitly described as a moral value or ethical principle;  solidarity is mentioned in a series with other moral values or ethical principles;  solidarity is described as belonging to the good;  Solidarity is mentioned in reference to concerns, considerations, decision, evaluations or failures;  or solidarity is taught through moral education. | |
| **Understanding 1**  Solidarity as a form of social cohesion is a condition for the implementation of CTA and their acceptance | **Understanding 2a**  The implementation and use of CTA *reinforce* existing or *create* new forms of social cohesion | **Understanding 2b**  The implementation and use of CTA *undermine* or *weaken* existing forms of solidary social cohesion | **Understanding 3**  Solidarity as a moral value *guides* or *orients* decisions and future actions concerning CTA | **Understanding 4**  Solidarity as a moral value *evaluates* past decisions and actions concerning CTA |
| Solidarity is described as condition for the implementation and acceptance of CTA;  solidarity promotes the acceptance and use of CTA;  or solidarity is necessary for the development of CTA. | The text explicitly states that CTA constitute or strengthen social cohesion;  CTA strengthen factors that are elementary for social cohesion;  CTA prevent negative consequences for the social cohesion;  or CTA open up possibilities of reimagining old and establishing new relationships. | The text explicitly states that CTA threaten, weaken or undermine social cohesion;  CTA weaken factors that are elementary for social cohesion or reinforce factors that damage social cohesion;  the introduction of CTA worsens the situation of a group of people or increases existing vulnerabilities or marginalizations;  or the resources used for the development and implementation of CTA would have had more positive effects on the community if used in an alternative way. | Solidarity is referred to as a central criterion for ethical or legal concerns, considerations or decision;  solidarity is introduced to orient or guide a decisions or actions;  solidarity imposes obligations to act;  or solidarity helps individuals to become aware of their role. | Solidarity refers to past discussions or decisions;  solidarity serves as criterion for the evaluation of past discussions or decisions;  solidarity is presented as a moral value with which past failures could have been avoided;  or solidarity is a criterion to evaluate other people’s behavior. |

Table 1. Illustration of the list of operators in Chapter 3.5 *Assignment of the papers to the five understandings*. The table the different operators that can be used to assign a mention of CTA in the text to an understanding. The key differences of whether solidarity is understood as a form of social cohesion and as a moral value as well as the different understandings of solidarity are highlighted in gray, below them and highlighted in white are the operators that can be used to assign a mention of solidarity to this key difference.

# Appendix 3

In the following table I will show which papers I have assigned to which understanding of solidarity in the context of contact tracing apps based on which operators. I will quote central parts of the papers that contain the notion solidarity and will underline relevant passages in these quotes. I will then show the operators I found in these passages that were decisive for assigning the paper to an understanding. Not all operators occur in the quoted parts, but are occasionally found in the context of the passage – if the quoted passage would otherwise have become too long. The table is arranged alphabetically according to the names of the first authors.

| **Name** | **Quote (relevant passages are underlined)** | **Operators** | **Understanding** |
| --- | --- | --- | --- |
| Alemanno, Bialasiewicz | “Third, they [primarily COVID-19 certificates but also contact tracing apps – MT] contribute to creating further forms of exclusion by appealing to a rhetoric of individual freedom and the ‘right’ to live untrammelled by COVID-19 restrictions – a rhetoric, as the authors observe, that risks undermining the collective response and solidarity necessary to combat the negative effects of the pandemic on communities: local, national or global.“ (284) | Solidarity describes the cohesion and cooperation of different actors; this solidarity is threatened and undermined by the introduction of discriminatory technologies (e.g., COVID-19 certificates and contact tracing apps). | 2b |
| Barocas et al. | “For example, when correlated with other data, the data collected by a contact-tracing project could be used to identify and persecute undocumented immigrants. Failure to guard against these risks will limit people’s willingness to rely on your project and may undermine the solidarity needed to maintain public health. Consequently, privacy and security must be paramount.” (32) | Solidarity refers to groups or individuals of social systems; the introduction of contact tracing apps worsens the situation of a group of people or increases existing vulnerabilities or marginalizations. | 2b |
| Batifoulier, Diaz-Bone | “Equality, solidarity, and responsibility are important values that governments have invoked as a way out of the crisis and as a way to reduce lockdown measures. […]. In a same way, the use of contact-tracing apps as a means in order to contain the spread of COVID-19 is not only a problem of temporary sacrifice of privacy because it is the conception of privacy that is the subject of different visions of the common good.” (25) | Solidarity is explicitly described as a moral value and is mentioned in a series with other moral values; solidarity a framework for ethical and legal concerns. | 3 |
| Blauth, Gstrein | “Measures for proximity and contact tracing, symptom checking, quarantine compliance, and flow modelling resonate in numerous social, ethical, and legal issues. These evoke challenges relating to individual and collective autonomy, justice, solidarity, beneficence, and non-maleficence, which need to be addressed throughout all stages of design, deployment, and evaluation of measures.” | Solidarity is mentioned in reference to challenges and in a series with other moral values; solidarity is referred to as a central criterion for these challenges. | 3 |
| Braun, Hummel | “For any given app, those answers will vary markedly, depending on whether the evaluation is primarily in terms of individual freedom, public health, solidarity with those most affected by the pandemic, the common good or some other ethical reference point.“ (360) | Solidarity is mentioned in a series with other moral values or ethical principles (freedom and public health); solidarity serves as criterion for the evaluation of past discussions or decisions. | 3 |
| Chang, Agliata, Guarinieri | “Materially, social distancing language and measures threaten to worsen the ongoing overdose crisis as peers are faced with contradictory public health messaging (never use alone/self-isolate) and community solidarity initiatives are being restricted.“ (2) | Solidarity denotes an organizational form of social systems; the introduction of contact tracing apps worsens the situation of a group of people (drug users). | 2b |
| Christofidou, Lea, Coorevits | “The authors discuss the importance that the EU has placed on solidarity, which has been recognised and encouraged by its citizens through the use of their data, as well as the prosocial motivations that governments have which appear through the governance models they have adopted. Discussions are made on the legitimacy of collecting this data and proposals are put forward on the need for transparency, verification and accountability to being the guiding principles, and the fulfilment of international obligations to collaborate and apply human rights.“ (230) | Solidarity is mentioned in a series with other moral values or ethical principles and in reference to evaluations on contact tracing apps; solidarity is presented as a criterion that other actors used to evaluate their decisions on contact tracing apps. | 4 |
| Dowthwaite et al. | “Trust may also significantly impact the adoption of contact  tracing apps […]. In Germany, it was also shown that general trust in official app providers as well as social trust played important roles, highlighting the importance of both data-securing issues and interpersonal solidarity.” | Solidarity describes the cooperation of different actors; solidarity is described as condition for the acceptance of contact tracing apps. | 1 |
| El-Haddadeh, Fadlalla, Hindi | While “[e]lucidating AI-Enabled Covid-19 Digital Proximity Tracking and Tracing” (4), the authors present a “Responsible AI Frameworks and Initiatives” (4). One of the four principles of this framework is solidarity: “The fourth principle of justice targets the upholding of prosperity and solidarity.” (5) | Solidarity is described as ethical principle and mentioned in a series with other ethical principles; solidarity is referred to as a central criterion in the framework for contact tracing apps. | 3 |
| Findlay, Remolina | “Recognise and comply with established principles of ethical AI, big data use, and principled design – Paramount among these principles for our purposes are  • Human dignity and solidarity when directed to individual liberty/integrity […]“ (9) | Solidarity is explicitly described as an ethical principle and is mentioned in a series with other ethical principles; solidarity is introduced to guide a future decision. | 3 |
| French et al. | “We wonder if the racial health disparities and injustices exacerbated by the COVID-19 pandemic could have been mitigated if the energy devoted to corporate contact tracing was instead channeled into improved access to healthcare and housing, workers’ protections, guaranteed incomes, and other measures designed to build social solidarity?” (6) | Solidarity implicitly refers to the group or poor individuals in a social system; the resources used for the development and implementation of contact tracing apps would have had more positive effects on the community if used in an alternative way. | 2b |
| Gasser et al. | “These four types of digital public health technologies raise ethical–legal considerations that are both cross sectional and domain-specific. These considerations are grounded in the basic principles and moral considerations of public health ethics and data ethics, in particular the principles of autonomy, justice, non-maleficence, privacy, and solidarity.” (3)  “The set of principles (beneficence, justice, nonmaleficence,  privacy, solidarity, and autonomy) discussed in this article can serve as a reference point.“ (6) | Solidarity is explicitly described as an ethical principle and is mentioned in a series with other ethical principles; solidarity is mentioned in reference to an ethical-legal consideration. | 3 |
| Georgieva, Beaunoyer, Guitton | “It is important to raise the fact that social acceptability is not static but dynamic, and can change according to the national and international context, and the balance of risks. That is why it is critical to consider all the decision-making process related to the implementation of technological tracking of the population with the necessary flexibility, and to understand that measures could be practically subjected to gradual changes. In the light of the various problems resulting from the differences between countries, the need for unification and solidarity is therefore crucial when discussing the changing nature of these decisions.“ (7) | Solidarity denotes an organizational form of social systems (countries); solidarity is described as one factor positively influencing the public acceptance of contact tracing apps and is therefore important to consider. | 1 |
| Gibney, Bruton, Doherty | “Moreover, participants suggested that the app would benefit from and foster solidarity among the public in the national ‘fight’ against the novel coronavirus.” (9) | Solidarity refers to groups or individuals of social systems (the public); solidarity promotes for the implementation and acceptance of contact tracing apps; *and* contact tracing apps strengthen solidarity. | 1  2a |
| Hendl, Chung, Wild | “Following on our discussion of vulnerability and broader debates on the ethical risks related to COVID-19 pandemic responses, it appears that beside the necessity of effectiveness and respect for privacy and confidentiality, COVID-19 apps ought to be explicitly grounded in values that include justice, equality, solidarity, and user-benefit.“ (4) | Solidarity is explicitly described as a moral value and is mentioned in a series with other moral values; solidarity refers to past discussions or decisions. | 4 |
| Hoffman et al. | “In this regard, future research into the ethical–political dimensions of Covid-19 contact tracing apps would be well served by continuing to unpack their (in)visibilities and the designerly normativities which propel decisions about what seams reveal and what shall be relegated to the realm of seamlessness. In particular, questions about what *other* values and user behaviors might be at stake in the design and use of such tools – such as solidarity or logics of care – are instructive to ask here.” (9) | Solidarity is explicitly described as a moral value and is mentioned in a series with other moral values; solidarity serves as criterion for the evaluation of past discussions or decisions *and* is mentioned in reference to future ethical decisions. | 3  4 |
| Hummel, Braun | “In the following, we contend that such an ethical reflection—not only on tracing apps, but also many other digital health applications—would need to address at least two topics: our pursuits of the good, of which acts of solidarity are paradigmatic instances, and the right, of which justice is one central component.“ (2) | Solidarity is described as belonging to the good; solidarity is mentioned in reference to ethical reflections. | 3 |
| Kahn | “Although privacy is a key value, individuals and communities may also value efficiency, equity, liberty, autonomy, economic well-being, companionship, patriotism, or solidarity, among other values.” (16) | Solidarity is mentioned in a series with other moral values; solidarity is introduced to guide the action of implementing contact tracing apps. | 3 |
| Kaspar | “Hence, when people have the impression that their fellow human beings are being less solidary by adhering less to recommended or even prescribed behaviors, they apparently attempt to compensate for this tendency by donating their personal data without receiving any direct counter value.” (10) | Solidarity is referred to as a central criterion for a decision; solidarity is a criterion to evaluate other people’s behavior. | 4 |
| Keating | “Narrative […] is a sequencing of events, a relationship to temporality and a conveyance of information that other humans often respond to with emotion, empathy or solidarity as they fall into the experience of hearing a story. […] Uneven success of devices and applications like the Google and Apple contact tracing application could have been avoided for a myriad of reasons: cultural, political, social and otherwise. But what if the designers had to read a novel” (20) | Solidarity is mentioned in reference to a past failure; solidarity is presented as a moral value with which the failure could have been avoided. | 4 |
| Lanzing | “Without privacy, citizens are all to a certain extent vulnerable to unwanted interference. It is therefore an act of solidarity to stand up for the right to privacy.“ (2)  “A contact tracing app that has not been designed and implemented based on public values, a democratic procedure and under strict conditions may undermine trust and solidarity in the long run.” (4) | Solidarity refers to groups or individuals of social systems (citizens); solidarity undermines factors that are elementary for social cohesion *and* increases existing vulnerabilities.  *And* solidarity explicitly described as a value; solidarity is a central criterion for a decision. | 2b  3 |
| Lee, Lee | “However, normalising behaviour encourages active and motivated participation by a population to fulfil loftier goals of national solidarity, and avoids the ‘politically costly phenomena of resistance and disobedience (developing) in the interstices’.” (56) | Solidarity refers to groups or individuals of social systems (population); contact tracing apps prevent negative consequences for their (national) solidarity. | 2a |
| Leslie | “At the negative extreme, this would mean that self-reinforcing mechanisms of social distrust end up optimizing privacy at the expense of creating conditions of deteriorated autonomy, social connectedness, and solidarity.” (13)  “[…] the potential for radically decentralized forms to reinforce social distrust and to harm individual autonomy and interpersonal solidarity—should perhaps draw our attention to an additional factor that must also be considered.” (14)  “The UK Government’s official public sector guide to safe and ethical AI has consolidated these into four ‘SUM values’—values that aim to support, underwrite, and motivate a responsible and reflective AI/ML innovation ecosystem and that are anchored in ethical concerns about human empowerment, interactive solidarity, individual and community wellbeing, and social justice.” (22) | Solidarity is explicitly described as a value and is mentioned in a series with other moral values; solidarity is mentioned in reference to ethical concerns.  *And* solidarity implicitly refers to a social system (society); contact tracing apps weaken factors that are elementary for social cohesion and reinforce factors that damage social cohesion. | 2b  3 |
| Lu et al. | When discussing contact tracing apps, among other “COVID-19 control programs”: “Social cohesion, which refers to the extent of connectedness and solidarity among groups in society, appears to be an important precondition for successful COVID-19 control programs.” (8) | Solidarity is explicitly described as social cohesion; solidarity is described as precondition for the implementation and acceptance of contact tracing apps | 1 |
| Mangan, Gramano, and Kullmann | “An unprecedented social solidarity stress test”  “[…]‘contact-tracing apps’ that would allow governments and citizens – employers included – to know if and when a certain individual was located in the proximity of an infected person.” | Solidarity describes the cohesion of a social system and solidarity refers to groups or individuals of social systems (governments, citizens); contact tracing apps weaken factors that are elementary for social cohesion (privacy, trust). | 2b |
| Matt | “The first group received the Societal Benefits treatment, with a persuasive message highlighting the tracing app’s benefits for protecting the entire society. The message further pointed out that using the app could help protect their families and friends, as well as elderly and disadvantaged individuals. It tried to appeal to the community, drawing on participants’ solidarity.” (6) | Solidarity describes a group of persons close to each other; the use of contact tracing apps is presented as something that could help the group, i.e., solidarity promotes the use of contact tracing apps. | 1 |
| Mbunge, Fashoto et al. | “the study proposes ethical framework for using emerging technologies to contain the COVID-19 pandemic. The framework is centered on ethical practices such as security, privacy, justice, human dignity, autonomy, solidarity, beneficence, and non-maleficence.” | Solidarity is mentioned in a series with other moral values or ethical principles; solidarity is referred to as a framework for ethical or legal concerns, considerations or decision. | 3 |
| Mbunge, Milham et al. | “[W]e analysed the available literature and propose an ethical framework for the use of digital technologies centred on ethical practices.” (190) Mbunge et al. consider solidarity as an ethical value among other ethical values (195) | Solidarity is mentioned in a series with other moral values or ethical principles; solidarity is referred to as one ethical value amongst other for a ethical or legal framework for contact tracing apps. | 3 |
| Mello, Wang | “It is aligned with both public health goals (because supported individuals are more likely to be able to stay home) and the principles of solidarity and reciprocity, which recognize societal obligations to support those called on to sacrifice liberty to prevent harm to others.” (953) | Solidarity is explicitly described as a principle and is mentioned in a series with another moral principle; solidarity imposes obligations to act. | 3 |
| Milan | “These solutions, including technology-based ones, might then serve ‘other’ needs such as the possibility to knit relationships of solidarity, care and mutual help and the creation of a nurturing community in times of emergency.” (6) | Solidarity refers to a social system (community); the resources used for contact tracing apps would have had more positive effects on the community if used in an alternative way. | 2b |
| Milne, Costa | “For example, contact tracing apps, such as those now implemented in France, Germany or South Korea, rely on adoption through a sense of solidarity, while their implementation, notably in China and Israel draws attention to the powers and capabilities of the state, while reinforcing these and raising significant and enduring concerns about surveillance and privacy.” (4) | Solidarity denotes an organizational form of social systems (countries); solidarity is described as condition for the implementation and acceptance of contact tracing apps. | 1 |
| Montanari Vergallo, Marinelli | “[…] one cannot blindly trust the exclusive help of technology. Instead, we need the involvement of health personnel, scientists, and no less importantly, the citizenry’s sense of solidarity and the duty to abide by the rules of social distancing, the use of protective devices and hygiene rules to protect public health.” (2449) | The citizenry’s sense of solidarity is presented as a sense for their interconnectedness and cohesion, i.e., their social cohesion; this sense of solidarity is presented as one factor promoting the use of contact tracing apps. | 1 |
| Nanni et al. | “Maintaining the trust of citizens at a time of crisis like the current one is a priority. […] Our proposal leverages both the respect for individual freedoms and for the environment, by cultivating feelings of solidarity and a sense of collective responsibility for rebuilding society.” (5) | Solidarity refers to a social system (an indeterminate society) and to a group of individuals in a social system (citizens); solidarity is described as condition for the implementation and acceptance of contact tracing apps. | 1 |
| Nijsingh, van Bergen, Wild | “Attempts to responsibly introduce CT technology are thus confronted with feedback loops: low effectiveness raises costs and decreases uptake, attempts to counter this by raising effectiveness may decrease privacy, which then potentially decreases uptake, while raising uptake by implementing more or less mandatory approaches creates risks of backlash and crumbling public support, which then again lowers effectiveness. Of course, scenarios where positive reinforcing feedback loops take place are also possible. A fair and reliable system would lead to an increase in trust and potentially a shared feeling of solidarity, which will lead to a further increase in use and therefore effectiveness, etc.” (3) | Solidarity implicitly refers to a social system (society); solidarity is described as condition for the implementation and acceptance of contact tracing apps *and* contact tracing apps strengthen factors that are elementary for solidarity *and* contact tracing apps weaken factors that are elementary for social cohesion or reinforce factors that damage social cohesion. | 1  2a  2b |
| Parker | “There are a number of reasons why those who have smartphones will have a strong incentive to sign up. The first of these is that this would ultimately mean that they and everyone else will emerge from the lockdown more quickly and safely. A second is that, by so doing, they will then be enabled to contribute to saving the lives of others, particularly the vulnerable, and those in caring roles, both locally and globally. Appeals to a sense of ‘we are all in this together’ of ‘solidarity’ may be effective.“ (3) | Solidarity refers to a group of individuals in a social system; solidarity promotes the implementation and acceptance of contact tracing apps. | 1 |
| Pila | “For example, and as Sandel has argued in respect of enhancement technologies, and with reference to medical insurance systems, too much information about individuals’ health risks and status can undermine solidarity by depriving  people of the very uncertainty about their own and others’ fates on which a commitment to sharing those fates depends. In this respect, contact tracing apps are similar to genetic and other enhancement technologies: by empowering individuals, they threaten the fabric of moral community.” (12) | Solidarity explicitly refers to a social system (moral community) and to a group or individuals in this social system; the text explicitly states that contact tracing apps undermine solidarity and threaten the moral community. | 2b |
| Price | “As the NHS Covid-19 app is only just being introduced, it does not have to be done so in a way that is business as usual. As Braidotti argues, we need ‘to embrace the opportunities offered by the new technologies and steer them towards new forms of solidarity and democratic debate and dissent’. We can use this opportunity to reimagine and reshape our relationship with technology from a social justice perspective.” (786) | Solidarity refers to groups or individuals (we); contact tracing apps open up the possibility of reimagining old and establishing new relationships. | 2a |
| Roche | “So, shouldn’t public authorities quickly open up a dialogue with the population, act as real educators, help citizens to feel safe and, make clear commitments regarding the risks […], but also help us to become aware of the important role that each of us plays in the big chessboard of urban social dynamics?” (14) | Solidarity is taught through moral education; solidarity helps individuals to become aware of their role. | 3 |
| Sagan et al. | “The declarations of the states of emergency have likely sparked a sense of threat but also of solidarity amongst the citizens, contributing to the high public acceptance of these measures […].” | Solidarity describes the cohesion amongst people; solidarity promotes the acceptance of contact tracing apps. | 1 |
| Samuel, Sims | “Our findings showed how, through the mixture of both promissory discourses and altruistic discourses of solidarity, an imaginary was created that was imbued with implicit understandings of what is good or desirable in the social world. The future-oriented visions and promises attached to the app, along with calls of social obligation, constructed the trial of the app as a venture which was morally good, which was valued because of its ability to bring health benefits, and which was desirable in the social world of the Isle of Wight.” (14) | Solidarity refers to a future form of social cohesion; contact tracing apps strengthen factors that are elementary for social cohesion.  Solidarity is described as belonging to the morally good; solidarity imposes obligations to act. | 2a  3 |
| Samuel et al. | “Corroborating other research, interviewees had a range of views about the use of digital contact tracing apps as part of the UK’s response to the COVID-19 pandemic. This ranged from those who were very supportive of using apps for contact tracing and spoke in terms of solidarity about using the data for research purposes for the benefit of all, to those who were extremely cautious about digital contact tracing apps.” (8) | Solidarity refers to the cooperation of different actors; solidarity is presented as an aspect that promotes the acceptance and use of contact tracing apps. | 1 |
| Saunes et al. | “There were also calls from the countries’ leaderships for solidarity to reduce the risk of infection spread and emphasis on voluntary effort, as all countries communicated a strong reliance on the public to follow the recommendations. Table 2 presents an overview of the main policy measures [among them: contact tracing apps – MT] adopted at population level in all the Nordic countries from March through August 2020.“ (5) | Solidarity describes the cohesion of a social system or an essential factor for it; solidarity is called for in order to ensure the acceptance and use of contact tracing apps | 1 |
| Sekalala et al. | “This context is important when it comes to digital public health surveillance mechanisms for COVID-19, since historical and contemporary rights abuses of surveillance mechanisms erode public trust and the solidarity necessary for the widespread adoption and public health efficacy of such mechanisms.” (8) | Solidarity implicitly refers to a social system (democratic society); solidarity is a condition for the implementation and acceptance of contact tracing apps *and* contact tracing apps erode factors that are elementary for social cohesion (public trust) and weaken solidarity. | 1  2b |
| Siffels | “Each order has its own conception of the common good, and in each order certain values are seen as more worthy than others. For example, in the civic order, the common good is conceptualized as acting for the good of society, and the values of solidarity, equality and inclusivity are foregrounded.” (2) | Solidarity is explicitly described as a value and is mentioned in a series with other values; solidarity serves as criterion for the evaluation of past ethical decisions. | 4 |
| Stefan | “This brings to the fore the criterion of inclusiveness and openness of the governance processes, with the introduction to the Communication stressing the importance of solidarity and ‘close cooperation among all relevant actors’ in tackling the pandemic.” (11) | Solidarity describes the cooperation of different actors; solidarity is necessary for the development of contact tracing apps. | 1 |
| van Hees et al. | “But digital health also gives rise to more complex forms of monitoring, blurring the boundaries between public and private surveillance, which could potentially lead to problems regarding autonomy (authenticity) and solidarity (e.g. poor surveillance of efficacy, safety and quality of the health app).” (14) | Solidarity denotes an organizational form of social systems (publicity and privacy); contact tracing apps threaten these organizational forms that are elementary for social cohesion. | 2b |
| Watson, Ali, Smeddinck | “It is recognised that technology considerations cannot be examined in isolation, and require people's trust in the systems being developed. The WHO suggests communications about contact tracing should ‘emphasize solidarity, reciprocity, and the common good‘ but recognises the need to be open about how information is used, stored, accessed, how people’s identity and data will be protected.“ | Solidarity is described as belonging to the good and is mentioned in a series with other moral values; solidarity serves as criterion for the consideration and examination of contact tracing apps. | 4 |
| Wnuk, Oleksy, Domaradzka | “For example, survivors of catastrophes behave more altruistically and exhibit enhanced solidarity towards each other. […] The above studies show that prosocial attitudes may be associated with a greater tendency to accept useful but potentially dangerous anti-COVID-19 technologies.” (2-3) | Solidarity describes the cohesion of a social system; solidarity promotes the acceptance of contact tracing apps. | 1 |

Table 2. Overview of the names of the authors of the papers, quotes from the papers containing the notion solidarity with the relevant passages underlined, the operators that can be found in the quotes, and the resulting understanding of the papers.

# References

Alemanno, A., & Bialasiewicz, L. (2021). Certifying Health: The Unequal Legal Geographies of COVID-19 Certificates. *European Journal of Risk Regulation*, *12*(2), 273-286. <https://doi.org/10.1017/err.2021.38>

Barocas, S., Biega, A. J., Boyarskaya, M., Crawford, K., Iii, H. D., Dudík, M., Fish, B., Gray, M. L., Hecht, B., Olteanu, A., Poursabzi-Sangdeh, F., Stark, L., Vaughan, J. W., Wallach, H., & Zepf, M. (2021). Responsible computing during COVID-19 and beyond. *Communications of the ACM*, *64*(7), 30-32. <https://doi.org/10.1145/3466612>

Batifoulier, P., & Diaz-Bone, R. (2021). Perspectives on the Economics and Sociology of Health. Contributions from the Institutionalist Approach of Economics of Convention – An Introduction. *Historical Social Research / Historische Sozialforschung*, *46*(1), 7-34.

Blauth, T. F., & Gstrein, O. J. (2021). Data-driven measures to mitigate the impact of COVID-19 in South America: how do regional programmes compare to best practice? *International Data Privacy Law*, *11*(1), 18-31. <https://doi.org/10.1093/idpl/ipab002>

Braun, M., & Hummel, P. (2020). Contact-tracing apps: contested answers to ethical questions. *Nature*, *583*(7816), 360. <https://doi.org/10.1038/d41586-020-02084-z>

Chang, J., Agliata, J., & Guarinieri, M. (2020). COVID-19 - Enacting a 'new normal' for people who use drugs. *Int J Drug Policy*, *83*, 102832. <https://doi.org/10.1016/j.drugpo.2020.102832>

Christofidou, M., Lea, N., & Coorevits, P. (2021). A Literature Review on the GDPR, COVID-19 and the Ethical Considerations of Data Protection During a Time of Crisis. *IMIA Yearbook of Medical Informatics*, 226-232. <https://www.thieme-connect.com/products/ejournals/pdf/10.1055/s-0041-1726512.pdf>

Dowthwaite, L., Fischer, J., Perez Vallejos, E., Portillo, V., Nichele, E., Goulden, M., & McAuley, D. (2021). Public Adoption of and Trust in the NHS COVID-19 Contact Tracing App in the United Kingdom: Quantitative Online Survey Study. *J Med Internet Res*, *23*(9), e29085. <https://doi.org/10.2196/29085>

El-Haddadeh, R., Fadlalla, A., & Hindi, N. M. (2021). Is There a Place for Responsible Artificial Intelligence in Pandemics? A Tale of Two Countries. *Inf Syst Front*, 1-17. <https://doi.org/10.1007/s10796-021-10140-w>

Findlay, M. J., & Remolina, N. (2020). Regulating Personal Data Usage in COVID-19 Control Conditions. *SSRN Electronic Journal*. <https://doi.org/10.2139/ssrn.3607706>

French, M., Guta, A., Gagnon, M., Mykhalovskiy, E., Roberts, S. L., Goh, S., McClelland, A., & McKelvey, F. (2020). Corporate contact tracing as a pandemic response. *Critical Public Health*, 1-8. <https://doi.org/10.1080/09581596.2020.1829549>

Gasser, U., Ienca, M., Scheibner, J., Sleigh, J., & Vayena, E. (2020). Digital tools against COVID-19: taxonomy, ethical challenges, and navigation aid. *The Lancet Digital Health*. <https://doi.org/10.1016/s2589-7500(20)30137-0>

Georgieva, I., Beaunoyer, E., & Guitton, M. J. (2021). Ensuring social acceptability of technological tracking in the COVID-19 context. *Computers in Human Behavior*, *116*. <https://doi.org/10.1016/j.chb.2020.106639>

Gibney, S., Bruton, L., & Doherty, P. (2020). *COVID Contact Tracing App: User Perspectives and Experience Research* (<https://igees.gov.ie/research-report-app-user-experience-and-perspectives-may-2020/>

Hendl, T., Chung, R., & Wild, V. (2020). Pandemic Surveillance and Racialized Subpopulations: Mitigating Vulnerabilities in COVID-19 Apps. *Journal of Bioethical Inquiry*. <https://doi.org/10.1007/s11673-020-10034-7>

Hoffman, A. S., Jacobs, B., van Gastel, B., Schraffenberger, H., Sharon, T., & Pas, B. (2020). Towards a seamful ethics of Covid-19 contact tracing apps? *Ethics Inf Technol*, 1-11. <https://doi.org/10.1007/s10676-020-09559-7>

Hummel, P., & Braun, M. (2020). Just data? Solidarity and justice in data-driven medicine. *Life Sciences, Society and Policy*, *16*(1), 8. <https://doi.org/10.1186/s40504-020-00101-7>

Kahn, J. P. (2020). *Digital Contact Tracing for Pandemic Response: Ethics and Governance Guidance*. J. H. U. Press

Kaspar, K. (2020). Motivations for Social Distancing and App Use as Complementary Measures to Combat the COVID-19 Pandemic: Quantitative Survey Study. *Journal of Medical Internet Research*, *22*(8), e21613. <https://doi.org/10.2196/21613>

Keating, J. (2020). Responsible Design Through Interdisciplinary Collaboration: Contact Tracing Apps, Surveillance and the Prospect of Building User Trust. *Bulletin of the Technical Committee on Data Engineering*, *43*(4), 11-22.

Lanzing, M. (2020). Contact tracing apps: an ethical roadmap. *Ethics and Information Technology*, 1-4. <https://doi.org/10.1007/s10676-020-09548-w>

Lee, T., & Lee, H. (2020). Tracing surveillance and auto-regulation in Singapore: ‘smart’ responses to COVID-19. *Media International Australia*, *177*(1), 47-60. <https://doi.org/10.1177/1329878x20949545>

Leslie, D. (2020). Tackling COVID-19 through responsible AI innovation: Five steps in the right direction. *SSRN Electronic Journal*. <https://doi.org/10.2139/ssrn.3652970>

Lu, G., Razum, O., Jahn, A., Zhang, Y., Sutton, B., Sridhar, D., Ariyoshi, K., von Seidlein, L., & Muller, O. (2021). COVID-19 in Germany and China: mitigation versus elimination strategy. *Glob Health Action*, *14*(1), 1875601. <https://doi.org/10.1080/16549716.2021.1875601>

Mangan, D., Gramano, E., & Kullmann, M. (2020). An unprecedented social solidarity stress test. *European Labour Law Journal*, *11*(3), 247-275. <https://doi.org/10.1177/2031952520934585>

Matt, C. (2021). Campaigning for the greater good? – How persuasive messages affect the evaluation of contact tracing apps. *Journal of Decision Systems*, 1-18. <https://doi.org/10.1080/12460125.2021.1873493>

Mbunge, E., Fashoto, S. G., Akinnuwesi, B., Metfula, A., Simelane, S., & Ndumiso, N. (2021). Ethics for integrating emerging technologies to contain COVID-19 in Zimbabwe. *Hum Behav Emerg Technol*. <https://doi.org/10.1002/hbe2.277>

Mbunge, E., Millham, R. C., Sibiya, M. N., Fashoto, S. G., Akinnuwesi, B., Simelane, S., & Ndumiso, N. (2021). Framework for ethical and acceptable use of social distancing tools and smart devices during COVID-19 pandemic in Zimbabwe. *Sustainable Operations and Computers*, *2*, 190-199. <https://doi.org/10.1016/j.susoc.2021.07.003>

Mello, M. M., & Wang, C. J. (2020). Ethics and governance for digital disease surveillance. *Science*, *368*(6494), 951–954. <https://doi.org/10.1126/science.abb9045>

Milan, S. (2020). Techno-solutionism and the standard human in the making of the COVID-19 pandemic. *Big Data & Society*, *7*(2). <https://doi.org/10.1177/2053951720966781>

Milne, R., & Costa, A. (2020). Disruption and dislocation in post-COVID futures for digital health. *Big Data & Society*, *7*(2). <https://doi.org/10.1177/2053951720949567>

Montanari Vergallo, G., Zaami, S., & Marinelli, E. (2021). The COVID-19 pandemic and contact tracing technologies, between upholding the right to health and personal data protection. *Eur Rev Med Pharmacol Sci*, *25*(5), 2449-2456. <https://doi.org/10.26355/eurrev_202103_25286>

Nanni, M., Andrienko, G., Barabasi, A. L., Boldrini, C., Bonchi, F., Cattuto, C., Chiaromonte, F., Comande, G., Conti, M., Cote, M., Dignum, F., Dignum, V., Domingo-Ferrer, J., Ferragina, P., Giannotti, F., Guidotti, R., Helbing, D., Kaski, K., Kertesz, J., Lehmann, S., Lepri, B., Lukowicz, P., Matwin, S., Jimenez, D. M., Monreale, A., Morik, K., Oliver, N., Passarella, A., Passerini, A., Pedreschi, D., Pentland, A., Pianesi, F., Pratesi, F., Rinzivillo, S., Ruggieri, S., Siebes, A., Torra, V., Trasarti, R., Hoven, J. V. D., & Vespignani, A. (2021). Give more data, awareness and control to individual citizens, and they will help COVID-19 containment. *Ethics Inf Technol*, 1-6. <https://doi.org/10.1007/s10676-020-09572-w>

Nijsingh, N., van Bergen, A., & Wild, V. (2020). Applying a Precautionary Approach to Mobile Contact Tracing for COVID-19: The Value of Reversibility. *Journal of Bioethical Inquiry*. <https://doi.org/10.1007/s11673-020-10004-z>

Parker, M. J., Fraser, C., Abeler-Dorner, L., & Bonsall, D. (2020). Ethics of instantaneous contact tracing using mobile phone apps in the control of the COVID-19 pandemic. *Journal of Medical Ethics*. <https://doi.org/10.1136/medethics-2020-106314>

Pila, J. (2020). Covid-19 and Contact Tracing: A Study in Regulation by Technology. *European Journal of Law and Technology*, *11*(2). <https://ejlt.org/index.php/ejlt/article/view/782>

Price, C. (2020). Covid-19: When Species and Data Meet. *Postdigital Science and Education*, *2*(3), 772-790. <https://doi.org/10.1007/s42438-020-00180-x>

Roche, S. (2020). Smile, you’re being traced! Some thoughts about the ethical issues of digital contact tracing applications. *Journal of Location Based Services*, *14*(2), 71-91. <https://doi.org/10.1080/17489725.2020.1811409>

Sagan, A., Bryndova, L., Kowalska-Bobko, I., Smatana, M., Spranger, A., Szerencses, V., Webb, E., & Gaal, P. (2021). A reversal of fortune: Comparison of health system responses to COVID-19 in the Visegrad group during the early phases of the pandemic. *Health Policy*. <https://doi.org/10.1016/j.healthpol.2021.10.009>

Samuel, G., Roberts, S. L., Fiske, A., Lucivero, F., McLennan, S., Phillips, A., Hayes, S., & Johnson, S. B. (2021). COVID-19 contact tracing apps: UK public perceptions. *Critical Public Health*, 1-13. <https://doi.org/10.1080/09581596.2021.1909707>

Samuel, G., & Sims, R. (2021). The UK COVID-19 contact tracing app as both an emerging technology and public health intervention: The need to consider promissory discourses. *Health (London)*, 13634593211060768. <https://doi.org/10.1177/13634593211060768>

Sekalala, S., Dagron, S., Forman, L., & Meier, B. M. (2020). Analyzing the Human Rights Impact of Increased Digital Public Health Surveillance during the COVID-19 Crisis. *Health and Human Rights Journal*.

Siffels, L. E. (2020). Beyond privacy vs. health: a justification analysis of the contact-tracing apps debate in the Netherlands. *Ethics Inf Technol*, 1-5. <https://doi.org/10.1007/s10676-020-09555-x>

Stefan, O. A. (2020). The Future of European Union Soft Law: A Research and Policy Agenda for the Aftermath Of COVID-19. *Journal of International and Comparative Law*, *7*(2), 329-350.

van Hees, S., Greubel, C., Moors, E., & Peine, A. (2021). Valuation in health and ageing innovation practices. *Ageing and Society*, 1-19. <https://doi.org/10.1017/s0144686x21001483>

Watson, C., Ali, R., & Smeddinck, J. D. (2021). Tensions and Mitigations: Understanding Concerns and Values around Smartphone Data Collection for Public Health Emergencies. *Proceedings of the ACM on Human-Computer Interaction*, *5*(CSCW2), 1-31. <https://doi.org/10.1145/3476071>

Wnuk, A., Oleksy, T., & Domaradzka, A. (2021). Prosociality and endorsement of liberty: Communal and individual predictors of attitudes towards surveillance technologies. *Computers in Human Behavior*, *125*. <https://doi.org/10.1016/j.chb.2021.106938>
